# Stages of Change, Smoking Behaviour and Readiness to Quit in a Large Sample of Indigenous Australians Living in Eight Remote North Queensland Communities

**DOI:** 10.3390/ijerph10041562

**Published:** 2013-04-16

**Authors:** Sandra Campbell, India Bohanna, Anne Swinbourne, Yvonne Cadet-James, Dallas McKeown, Robyn McDermott

**Affiliations:** 1 of Health Sciences, University of South Australia, Adelaide 5000, South Australia; E-Mail: Robyn.McDermott@unisa.edu.au; 2 of Public Health, Tropical Medicine & Rehabilitation Sciences, James Cook University, Cairns, Queensland 4870, Australia; E-Mail: India.Bohanna@jcu.edu.au; 3 of Psychology, James Cook University, Townsville, Queensland 4810, Australia; E-Mail: anne.swinbourne@jcu.edu.au; 4 of Indigenous Australian Studies, James Cook University, Townsville, Queensland 4810, Australia; E-Mail: yvonne.cadetjames@jcu.edu.au; 5 Health Service, Atherton, Queensland 4883, Australia; E-Mail: DMcKeown@wuchopperen.com

**Keywords:** Indigenous Australians, smoking, stages of change

## Abstract

Tobacco smoking is a major health issue for Indigenous Australians, however there are few interventions with demonstrated efficacy in this population. The Transtheoretical Model may provide a useful framework for describing smoking behaviour and assessing readiness to quit, with the aim of developing better interventions. Interviews were conducted with 593 Indigenous Australians in eight rural and remote communities in north Queensland, to examine stages of change and smoking behaviour. Among current smokers, 39.6% and 43.4% were in Precontemplation and Contemplation stages respectively. A further 13.9% were making preparations to quit (Preparation) whilst only 3.2% said they were actively trying to quit (Action). When analysed by stage of change, the pattern of smoking-related behaviours conformed to the results of past research using the model. Importantly however, distribution of individuals across the stages opposes those observed in investigations of smoking behaviour in non-Indigenous Australian populations. The Transtheoretical Model can be used to meaningfully classify Indigenous smokers in remote north Queensland according to stages along the behaviour change continuum. Importantly, in this large sample across eight communities, most Indigenous smokers were not making preparations to change their smoking behaviour. This suggests that interventions should focus on promoting movement toward the Preparation and Action stages of change.

## 1. Introduction

While Australia has some of the most extensive tobacco control measures in the Western world [1], Indigenous Australians do not share the same low rates of tobacco smoking as non-Indigenous Australians. The most recent national surveys indicate that 47% of Indigenous Australians smoke tobacco [2], compared with 17% in the general population [3]. In regional and remote Indigenous communities smoking rates may be higher still, with reports from the Well Persons Health Check and other surveys conducted in remote communities indicating tobacco smoking rates up to 77% [4,5,6,7,8,9,10]. The natural history of smoking also differs between the Indigenous and non-Indigenous Australian population. Indigenous smokers start younger [11], are more likely to develop smoking related conditions and are more likely to die from smoking related conditions than non-Indigenous smokers [12,13,14]. 

Considering these statistics, it is unsurprising that tobacco smoking represents the major risk to life and health within Indigenous Australian communities, with high smoking rates reflected in patterns of mortality and morbidity [14,15]. Between 1989 and 1999 the smoking-related death rate amongst Indigenous Queenslanders was almost three times greater than that of non-Indigenous Queenslanders [13]. Tobacco smoking is also a major contributor to low infant birth weight and complications arising from diabetes mellitus, cardiovascular disease and respiratory problems [12,13].

Despite these high rates of tobacco smoking in Indigenous individuals, there are few smoking cessation interventions conducted in this population, with limited evidence of efficacy [4,16,17,18,19,20]. In the context of tobacco control interventions, the Transtheoretical Model proposed by Prochaska and DiClemente and colleagues [21,22] lends itself to the examination of smoking behaviour change with a view to developing “stage matched interventions”. Briefly, the model proposes that the change process can be conceptualised into five stages: Precontemplation, Contemplation, Preparation for Action, Action and Maintenance. Individuals are hypothesised to move linearly through these stages and may relapse to earlier stages. Each stage is characterised by the presence of divergent beliefs about, knowledge of and attitudes towards the target behaviour. The model may assist in determining the type and range of interventions required; matching interventions to an individual or group’s current stage may increase their likelihood of causing positive behaviour change [23].

There is a lack of data addressing readiness to change smoking behaviour within Indigenous communities. One study examined stages of change and smoking behaviour in 66 Indigenous pregnant women, reporting 32%, 55% and 13% in Precontemplation, Contemplation, and Action stages respectively [24]. Another study of 50 Indigenous women reports that “the majority of women were “pre-contemplative” however no stage percentages are reported” [25]. Neither study reports smoking behaviours or knowledge by stage. The aim of this study was to apply the Transtheoretical Model to describe smoking behaviour in a large sample of Indigenous Australians living in remote north Queensland communities. In particular, the study sought to examine the relationship between stage of change and smoking behaviour. It was anticipated that this may assist in developing and implementing smoking cessation interventions in north Queensland Indigenous Australian populations.

## 2. Experimental Section

### 2.1. Recruitment and Sampling

This study formed part of a larger intervention study aimed at reducing tobacco use in north Queensland Indigenous communities. Baseline household surveys were conducted in eight rural and remote communities between October 2003 and March 2004. Communities ranged from approximately 200 to 1,000 people. All communities were consulted over a period of several months and were invited to participate if their population was predominately Indigenous Australian, there was a willingness to undertake tobacco control programs and they possessed the required infrastructure to participate in the broader trial (*i.e*., a health service, a school, and a community store). One community approached declined to participate.

Face-to-face interviews were conducted with individuals in 475 households. Census data in these communities systematically underestimates the population [26], therefore a sampling strategy based upon the number of permanent households in each community was employed. In the six communities with less than 70 households, every house was approached to participate. In the two larger communities, two out of three households were selected sequentially for interview based on maps provided by local councils. If residents at one of the households declined, or were not at home, the third household was approached. Over the eight communities, 702 individuals were surveyed. Almost 85% (n = 593) reported identifying as Indigenous Australians and were included in this analysis. 

### 2.2. Survey Instrument & Administration

The survey instrument included questions from national and state standardised surveys of smoking behaviour. The instrument was trialed with a small number of Indigenous volunteers for appropriateness of phrasing and required literacy competency and amendments were made in line with feedback. Questions were in plain English. To assess position along the “stage of change” continuum, questions were developed using the classification algorithms described by DiClemente *et al.* [22].

The final instrument consisted of primarily closed answer questions in which individuals selected the most appropriate answer from a list of possible responses (ranging in number from two to eight). Demographic data, frequency of smoking and number of cigarettes smoked per week (calculated from self-reported daily smoking) was assessed, along with the length of time the individual had used tobacco. Smokers were asked whether they planned to give up smoking in the future and whether they had attempted to quit or cut down in the last 12 months. Respondents who stated they had tried to quit or cut down were asked to indicate the type of event or information that prompted their actions. Respondents were asked about the smoking policy within their workplace and if smoking was tolerated inside their home and about their use or otherwise of smoking cessation pharmacotherapies.

Indigenous interviewers were recruited from each community and received training in the administration of the interview. Each interviewer was allocated a number of households to visit with the aim of interviewing two individuals >18 years of age from each household. Within each household, residents nominated the individuals to be interviewed. Given the low literacy levels in this region, information about the nature and purpose of the survey was read out from a printed form by the interviewer. Signed consent was obtained prior to interview. The study was approved by the Ethics Committee of James Cook University with the support of the relevant Indigenous community health councils.

### 2.3. Data Analysis

All analyses were conducted using SPSS 11.0. All tests of associations between categorical variables were conducted using χ^2^ tests. Where appropriate χ^2^ tests for trend were conducted. Tests of differences between means were conducted by ANOVA with post hoc Tukey tests. 

### 2.4. Stage of Change Classification

Individuals who reported smoking were asked if they were planning to give up. Following the classification algorithms described by DiClemente *et al.* [22], individuals were allocated to one of the following self-defined stages of change: Precontemplation (currently smoking and not planning to give up), Contemplation (currently smoking with a desire to give up smoking but not in the next month), Preparation for action (currently smoking and planning a quit attempt in the next month), Action (had smoked in the past year but were no longer smoking), Maintenance (had smoked sometime in the past but not in the previous year).

## 3. Results and Discussion

### 3.1. Results

#### 3.1.1. Characteristics of Sample

The response rate in communities ranged from 53% to 88% of targeted households, with mean and median response rates of 76% and 79% respectively. More young females agreed to be interviewed than young men. Table 1 shows the breakdown of demographic characteristics by ethnicity. The sex distribution did not differ over ethnic groups (χ^2^_2 _= 0.66, *p* = 0.72) however those individuals identifying as being of both Aboriginal and Torres Strait Islander descent were significantly younger on average than those identifying as either Aboriginal (*p* ≤ 0.01) or Torres Strait Islander (*p* ≤ 0.01). The mean (SD) number of adults per household was 2.9 (1.4). The distribution of minors in households was similar with an average of 2.2 (1.9). Approximately 60% of the cohort was in paid employment. For these individuals, hours worked ranged from 2–80 h a week, with half working 34 h or less a week. On average, the number of hours a week of paid employment within the sample was 29.1 (12.6). Of those in paid employment, 61% indicated that they were aware of a smoking policy in operation at their place of employment.

**Table 1:** Demographic characteristics of study participants (n = 593).

	Aboriginal	Torres Strait Islander	Aboriginal and TS Islander
Number (% *)	217 (36.6)	228 (38.4)	148 (25.0)
Number (% ^†^) women	129 (59.4)	144 (63.2)	90 (60.8)
Mean (range) age (years)	38.3 (16–84)	40.0 (18–87)	33.8 (18–78)

*: indicates percent of total sample, **^†^**: indicates percent of ethnic group.

#### 3.1.2. Smoking Behaviour

Of the sample, 74.8% (n = 444) reported that they had smoked tobacco at some time in their lives. Of those who had ever smoked, 61.7% reported that they currently smoked every day, 11.1% said that they smoked at least once a week and 6.8% reported smoking less than once a week but within the last year. The remaining 20.5% of participants reported that they had not smoked in the last year. The average age (SD) at which individuals reported smoking their first full cigarette was 16.9 (4.2) years (median 16 years). Age of smoking the first full cigarette did not differ between males and females (95% CI for the difference, −0.58–1.0, *p* = 0.6). The percentage of males reporting ever smoking was slightly but not significantly higher than that of females (78.5% and 72.5% respectively, 95% CI for difference, −0.012–0.129, *p* = 0.1). Employment status was not related to ever having smoked (χ^2^_1_ = 0.17, *p* = 0.7 and current smoking behaviour was independent of sex (χ^2^_4_ = 1.3, *p* = 0.87) and employment status (χ^2^_4_ = 2.4, *p* = 0.66).

#### 3.1.3. Stage of Change

Table 2 shows the breakdown of the sample and smoking behaviours by stage of change for those currently smoking. Of these, 20.5% (89 individuals) who had ever smoked and quit (*i.e.*, not smoked in the last year) were allocated to the Maintenance stage. The results in Table 2 are based on 346 individuals who reported smoking in the past year. Nine smokers who reported smoking in the past year were not able to be classified to a stage due to missing data. Stage of change was independent of sex (χ^2^_4_ = 4.5, *p* = 0.34) and employment status (χ^2^_4_ = 2.7, *p* = 0.61). Those in the Maintenance stage were significantly older (mean 44.7 years, 95% CI, 41.1–48.2) than those in any other stage. No mean age differences were detected between any other stages (which ranged from 28 to 37.9 years). 

For those currently smoking, smoking history differed little across stages of change. The length of time individuals reported that they had smoked on a daily basis decreased across stages, however this variable did not differ significantly between stages. The one difference detected was that individuals in the Maintenance stage reported having smoked daily in the past for significantly longer than those in any other stage (mean years 28.5, 95% CI, 23.5–33.4), however this may be due to this group being significantly older than any other group and basically having had more years in which to smoke. The age at which individuals had their first full cigarette did not differ between stages. 

**Table 2:** Smoking behaviour, intake and quit attempts of current smokers categorised by stage of change.

	Precontemplation	Contemplation	Preparation	Action
% of current smokers (n)	39.6 (137)	43.4 (150)	13.9 (48)	3.2 (11)
Mean (95% CI) age (years)	37.9 (35.5–40.4)	35.0 (32.9–37.2)	33.3 (30.3–36.2)	28 (21.4–34.6)
Age (95% CI) at first full cigarette	16.4 (15.8–17.0)	17.0 (16.3–17.7)	16.2 (15.3–17.2)	16.2 (14.4–18.0)
Every day% (n)	87.6 (120)	78.7 (118)	68.8 (33)	0 (0)
At least once a week% (n)	8.0 (11)	14.7 (22)	25.0 (12)	9.1 (1)
Less than once a week% (n)	2.9 (4)	4.7 (7)	6.3 (3)	9.1 (1)
Hardly ever, but in the last year% (n)	1.5 (2)	2.0 (3)	0 (0)	81.8 (9)
Number of cigarettes smoked per week ^+^	112.6 ± 73.4	96.8 ± 78.2	78.0 ± 81.8	0.4 ± 1.2
Years (95% CI) smoked every day ^+^	19.2 (16.6–21.7)	15.7 (13.4–17.9)	13.7 (10.3–17.0)	11.3 (0.9–21.8)
Number of attempts to quit or cut down in the last year ^+^	1.2 ± 1.3	1.9 ± 1.2	2.1 ± 1.0	1.4 ± 1.8
Cut down cigarettes in the last year *	40.2	52.3	73.8	100

*: As percent of stage. **^+^**: Mean. ±: One standard deviation.

While most individuals in the Precontemplation, Contemplation and Preparation stages reported smoking every day, this proportion decreased with proximity to the Action stage. This pattern was reversed for smoking frequencies of once a week or less (χ^2^_1_ for trend = 47.9, *p* < 0.01). The number of cigarettes smoked per week varied significantly across stages. While no difference in the average number of cigarettes smoked a week was detected between the Precontemplation and Contemplation stages (*p* = 0.68), individuals in these stages smoked significantly more on average than those in the Preparation stage (*p* = 0.002 and 0.010, respectively), who in turn smoked more than those in the Action phase (*p* = 0.036). 

Respondents were asked if they had attempted any of six ways to cut down their tobacco consumption. Individuals in the Precontemplation stage made significantly fewer attempts to cut down on their smoking than those in the Contemplation or Preparation stages (Table 2) (*p* < 0.001). The number of attempts made by those in the Preparation stage did not differ to that reported by individuals in the Contemplation stage (*p* = 0.398). Values for the Action phase did not differ significantly from any other phase, however this may reflect the low smoking frequency in that group. All those in the Action phase reported that they had successfully cut down their smoking in the past year (Table 2). The percentage of individuals reporting some success in cutting down varied from 40% of individuals in Precontemplation to nearly 75% of those in the Preparation stage (χ^2^_1_ for trend = 12.2, *p* < 0.001). Of those who reported smoking in the past year, 28 (8.1%) had used nicotine replacement therapy (patches or gum) and 6 individuals (<2%) said that they had been prescribed an oral smoking cessation medication (Zyban—bupropion hydrochloride).

### 3.2. Discussion

The current study aimed to use the Transtheoretical model to describe the tobacco smoking behaviour of Indigenous Australian individuals living in remote north Queensland communities. Our findings indicate that it is possible to classify Indigenous smokers in remote north Queensland into the stages of change, as proposed by the Transtheoretical model. Current smoking habits at each stage of change were as expected, based on earlier investigations using the model [22,27] with individuals in the earlier stages smoking significantly more cigarettes than individuals in later stages, and more attempts to quit or cut down being made by individuals in the Contemplation and Preparation stages compared with those in the Precontemplation stage. As in earlier reports, for those who had smoked in the past year, no relationship between age, sex and stage of change was detected [22,27]. Smoking history also bore no significant relationship to where an individual stood along the stage of change continuum which is similar to findings previously reported by DiClemente *et al.* [22]. 

We identified rates of smoking that were much higher than rates in non-Indigenous Australians, with fewer smokers planning to quit. In the present sample 62% of those who had ever smoked were currently smoking every day. Importantly for planning future tobacco cessation interventions in north Queensland Indigenous communities, 80% of the Indigenous Australians surveyed were in Precontemplation or Contemplation stages, with only 3.2% of the current smokers indicating they were actively trying to give up. Comparison data for stage of change classification was sourced from an investigation conducted in an Australian metropolitan region [27] where telephone and face-to-face household interviews were used to ask individuals about smoking, among other health related behaviours. Allocation to stage of change in the metropolitan study used similar classification algorithms as presented above, but with a time frame of one fortnight rather than one month. The percentage in the Precontemplation stage in our study was nearly double that reported in the comparison study (39.6% *vs.* 20.8% respectively). Similarly a larger percentage of smokers in the current study was in the Contemplation stage (43.4% *vs.* 29%). 

Importantly, the difference in smoking rates does not explain the different distribution we observed across the stages of change. Prochaska and DiClemente [21] argue that progression through the stages requires changes in knowledge, attitudes and beliefs about a behaviour. For example to move from Precontemplation to Contemplation an individual must know, at the very least, that the behaviour is detrimental to their health. National research undertaken during 2001 by the National Aboriginal and Torres Strait Islander Tobacco Control Project found that over 90% of participants agreed that smoking caused a range of serious illness [28]. However, knowledge of smoking related health damage does not necessarily lead to a desire to quit [29]. High rates of tobacco use in Australian Indigenous communities are also likely to be grounded in historical influences and cultural factors combined with personal circumstances that may include “habit, boredom, addiction, anxiety and depression” [29]. There appear to be barriers to smoking cessation other than poor health literacy among participants in this study, with more individuals being in the Precontemplation or Contemplation stages. 

Access to effective cessation aids may also have contributed to the low percentage of the present sample in the Action stage. Only 28 (8.1%) of those who had ever smoked had ever used nicotine replacement therapy (patches or gum). At six individuals, the number using Zyban was even lower. Movement from Contemplation to later stages requires knowledge about how to change one’s behaviour along with access to strategies and resources that would facilitate the change. It may therefore be that lack of access to nicotine replacement therapy also contributed to the low proportions at later stages within the present sample. In 2009, Clough *et al.* reported that 40% of 133 smokers knew nothing about pharmacotherapies, and 47% knew about them but had never tried them. Only 14% had used pharmacotherapies to quit, less than half the rate in non-Indigenous Australian smokers trying to quit [6,30]. From 2008, under the Council of Australian Governments Closing the Gap National Health Partnership, the national, state and territory governments have made significant investments in tackling smoking among Indigenous Australians [10]. Activities during 2010–2011 have included the rollout of smoking cessation and reduction programs which may result in as yet unmeasured health gains [31].

A limitation of this study is that the data was acquired in 2004, however, the study is likely to have contemporary relevance because of the on-going high smoking rates reported in Australian Indigenous communities [11]. In 2008 in remote Queensland, not only were smoking rates high, Indigenous adults were more likely to be current smokers (58% for males and 47% for females) than Indigenous adults living in rural and urban settings [10]. Since completion of this study, as well as the considerable investment in tobacco control initiatives for Indigenous Australians, general Australian government policy developments have included increases in tobacco taxes, the introduction of plain packaging of tobacco products and expansion of regulations defining smoke-free areas. The authors are unaware of any studies of a similar scale that have more recently assessed stages of change or smoking behaviour in a similarly large sample of Indigenous Australians. Importantly, this study provides baseline data for comparison with future work and offers valuable information and insight into an area with a paucity of evidence. The study is also limited by the small numbers of people in the later stages of change (Preparation and Action) which may have affected the power of the study to detect differences between stages. Finally, the study has relied on self-report of smoking habits which may have introduced a response bias related to sensitivities associated with the behaviour.

## 4. Conclusions

The current study investigated smoking behaviour and intentions to quit, classifying remote north Queensland Indigenous smokers into stages along the behaviour change continuum. Most smokers were not considering cutting down, suggesting that interventions for smoking cessation should promote progression from Precontemplation and Contemplation toward Preparation and Action.
